# *In Vivo* Anti-*Vibrio* Evaluation of Sponge-Associated Bacteria on the Survival Rate of *Litopenaeus vannamei* Infected with Pathogenic *Vibrio* Species

**DOI:** 10.21315/tlsr2023.34.2.15

**Published:** 2023-07-21

**Authors:** Jepri Agung Priyanto, Galuh Adhiyaksa Ashari, Munti Yuhana, Aris Tri Wahyudi

**Affiliations:** 1 Division of Microbiology, Department of Biology, Faculty of Mathematics and Natural Sciences, IPB University, Bogor 16680, Indonesia; 2 Department of Aquaculture, Faculty of Fisheries and Marine Sciences, IPB University, Bogor 16680, Indonesia

**Keywords:** Sponge-Associated Bacteria, Survival Rate, Vibriosis, *Litopenaeus vannamei*

## Abstract

Sponge-associated bacteria are considered a rich source of bioactive compounds particularly to reduce the risk of *Vibrio harveyi* and *Vibrio parahaemolyticus* infection. The present study aimed to analyse the effectiveness of 19 isolates to control *Vibrio* infection *in vivo*. All 19 isolates displayed a non-pathogenic characteristic on shrimps (cell density of 10^6^ cells/mL) as analysed using the pathogenicity test. The mortality caused by both *Vibrio* spp. on 50% of the shrimp population (LC_50_ value) had a cell density of 10^5^ cells/mL as determined using the proportion interval method. On the basis of the challenge test, all isolates improved the survival rate of infected shrimps in diverse effectivities up to 89%, which was nearly 30% higher than the infected control. Two isolates coded as D6.9, and P5.20 reduced shrimp mortality after infection with *Vibrio* spp. 16S rRNA-based identification showed these isolates were closely similar to different genera of *Bacillus* and *Staphylococcus*. The extract derived from the most prospective isolate, D6.9, was dominated by 1-hydroxy-6-(3-isopropenyl-cycloprop-1-enyl)-6-methyl-heptan-2-one, hexadecanoic acid, 4-epicyclomusalenone [(24S)-24-methyl-28-norcycloart-25-en-3-one], and 2,4-dimethyl acetoacetanilide. This observation suggested these isolates characterised by *in vivo* anti-*Vibrio* activity need to be further developed as biocontrol candidates.

**Highlights**

- 19 strains of sponge-associated bacteria improved the survival rate of *Vibrio*-infected shrimps.
- Two selected isolates (D6.9 and P5.20) was identified as *Bacillus aerius* and *Staphylococcus cohnii*.
- Secondary metabolite extract derived from the most prospective isolate (D6.9) contained hexadecanoic acid, which is known as antimicrobial compound.

## INTRODUCTION

Shrimp farming, mainly of *Litopenaeus vannamei* culture, is one of the industries that contribute a major income to several countries. In 2016, most shrimp production (87%) happens in Asia (Food and Agriculture Organization [FAO] 2016), including Indonesia (Wijayanto *et al*. 2017), India (Gunalan *et al*. 2014), and China (FAO 2016). Shrimps serve as an important source of nutrition and income because of their richness in protein content and rapid growth, respectively (FAO 2016). Unfortunately, an outbreak of infectious diseases hampers shrimp farming yield. Vibriosis is a major bacterial infectious disease caused by *Vibrio* spp. *Vibrio harveyi* and *Vibrio parahaemolyticus* are known as causative agents of the acute hepatopancreatic necrosis disease (AHPND), one of the types of vibriosis diseases, infected *L. vannamei*. The main symptoms of the disease include atrophied and pale hepatopancreas (HP), and an empty gut (Muthukrishnan *et al*. 2019). The disease is responsible for a high rate of mortality and morbidity, with the consequence of a huge economic loss (Srinivas *et al*. 2016). To effectively control vibriosis on shrimps, an ecofriendly treatment control is required.

Currently, microbial-based aquaculture treatments through both microbial metabolite-based products (antibiotics) and living cell-based products (probiotics) are considered susceptible treatments of the infectious disease. A diverse antibiotic product is widely used by shrimp farmers to control vibriosis, but the situation is aggravated by the emergence of antibiotic-resistant strains. Lee *et al*. (2018) reported that more than 50% of *V. parahaemolyticus* strains isolated from freshwater and fish surprisingly presented high resistance to amikacin (64%), ampicillin (88%), and kanamycin (50%). Therefore, discovery of new antibiotics is still an intriguing research interest to solve that problem. In addition, as the replacement for antibiotic treatments, probiotics are considered an effective way to increase shrimp aquaculture productivity by improving shrimp intestinal microbial balance and the inhibition of pathogenic bacterial growth (Jamal *et al*. 2019). Therefore, the selection of beneficial bacteria as both a probiotic candidate and an antibiotic producer is an urgent need.

Marine bacteria associated with sponges are regarded as a promising source of microbial bioactive compounds (Prastya *et al*. 2020; Priyanto *et al*. 2022). Some antimicrobial compounds, such as kocurin (Palomo *et al*. 2013), subtilomycin (Phelan *et al*. 2013), bacteriocin (Azemin *et al*. 2015), macrolactin A (Riyanti *et al*. 2020), amifloxacin and fosfomycin (Bibi *et al*. 2020), have been reported to be produced by marine sponge-associated bacteria. In our previous studies (Wahyudi *et al*. 2018; 2019), 19 bacterial isolates were successfully isolated from seven species of Indonesian sea sponge (*Spongia* sp., *Svenzea* sp., *Ircinia* sp., *Igernella* sp., *Hyrtios* sp., *Verongula* sp. and *Smenospongia* sp.) and selected according to their anti-*Vibrio* activity against *V. parahaemolyticus*, *V. harveyi* and *V. vulnificus*. These isolates showed a potent anti-*Vibrio* property *in vitro* with different spectra from broad to narrow spectra. Of the 19 isolates used in this experiment, eight isolates could inhibit both *V. harveyi* and *V. parahaemolyticus*, but the other 11 isolates could inhibit only one *Vibrio* species, either *V. harveyi* or *V. parahaemolyticus*. However, an *in vivo* investigation of that activity to control *Vibrio* growth and infection is required to evaluate the effect of these bacteria in controlling shrimp larvae mortality. The present study aimed to evaluate the non-pathogenicity of 19 bacterial isolates on shrimps and investigate the effect of these bacteria on the survival rate of shrimp larvae infected with *V. parahaemolyticus* and *V. harveyi*. The most promising isolate and its derived extract were then identified.

## MATERIALS AND METHODS

### Bacterial Isolates and Shrimp Sources

The bacteria used were *V. parahaemolyticus* ATCC 17802 (collected from the Laboratory of Fish Quarantine Standard Quality Test Control and Fishery Safety, Indonesia), and *V. harveyi* P-275 (collected from the Research and Development Laboratory of Brackish Aquaculture Water, Maros, Indonesia). Nineteen isolates of sponge-associated bacteria were collected from our previous studies (Wahyudi *et al*. 2018; 2019) and cultured in seawater complete medium (SWC) (composition: 750 mL of seawater, 250 mL of distilled water, 5 g of peptone, 3 mL of glycerol, and 1 g of yeast extract). Post-larvae 10 (PL-10) *Litopenaeus vannamei* (Pacific white shrimp) came from PT. Suri Tani Pemuka hatchery Indramayu-West Java and Pandeglang-Banten, Indonesia.

### Pathogenicity Assay of Bacterial Isolates on Shrimps

Pathogenicity assay was conducted by following Rini *et al*. (2017), with some modification. The assay was conducted in an aquarium containing 3 L of seawater and 10 individual PL10 *L. vannamei*. Nearly 10^6^ cells/mL of each bacterial suspension (cultured in SWC medium) were inoculated to the aquarium. The aquarium inoculated with respective *V. harveyi* and *V. parahaemolyticus* (10^5^ cells/mL) served as the positive control. The uninoculated aquarium was used as the negative control. The shrimps were fed with *Artemia salina* larvae (three times in a day). Each treatment was performed in triplicates. After 7 days, the mortality of shrimp larvae was counted and calculated as the percentage of survival rate.

### Determination of Lethal Concentration 50 (LC_50_) of *V. harveyi* and *V. parahaemolyticus*

The determination of LC_50_ value was carried out in an aquarium containing 3 L of seawater and 15 individual PL10 shrimps. Each aquarium was then inoculated with respective *Vibrio* suspension with various cell densities from 10^3^ cells/mL to 10^6^ cells/mL. Each treatment was performed in triplicate. The shrimps were fed with *A. salina* larvae (three times in a day). After 7 days, the mortality of shrimp larvae was tabulated. The survival rate of shrimps was converted into the mortality ratio. The LC_50_ value was then calculated using the following formula:


$$\text{Proportional interval}=\frac{\text{Percentage of the mortality more than }50\%-50}{\text{Percentage of the mortality more than }50\%-\text{Percentage of the mortality less than }50\%}$$

The proportion interval value was then used for the calculation of the negative log of the LC_50_ value. The negative log of the LC_50_ value is defined as the sum of the negative log of *Vibrio* cell density, which caused more than 50% shrimp mortality, and the proportion interval value (Reed & Muench 1938).

### *In Vivo* Challenge Assay on PL10 *L. vannamei*

A challenge test was carried out by inoculating each bacterial candidate suspension (10^6^ cells/mL) to the aquarium containing 3 L of seawater and 15 individual shrimps. After 6 h, *Vibrio* suspension (10^5^ cells/mL), including *V. harveyi* and *V. parahaemolyticus*, was inoculated into the aquarium (without sponge-associated bacteria candidates) and served as an infected control, whereas the aquarium without bacterial inoculation served as the untreated control. Each treatment was performed in triplicates. The shrimp culture was fed with *A. salina* three times in a day. The mortality of shrimps was then counted on the seventh day. The survival rate of shrimps was calculated using the following formula:


$$\text{Survival rate }(\%)=\frac{\text{Nt}}{\text{No}}\times 100$$

where, survival rate (SR) = the percentage of survival rate (%); Nt = the number of living shrimp larvae at the end of treatment (individual); No = the number of living shrimp larvae at the beginning of treatment (individual).

### 16S rRNA Gene-Based Identification of Two Selected Isolates

The potential isolates were cultured on Luria–Bertani broth medium (composition: 1 g of tryptone, 0.5 g of yeast extract, and 1 g of NaCl). Genomic DNA of 24 h culture was then extracted using the Genomic DNA Mini Kit (Blood/Cultured Cell, Geneaid, Taiwan) according to the manufacturer’s instruction. The universal primers of 1387R primer (5′-GGG CGG WGT GTA CAA GGC-3′) and 63F primer (5′-CAG GCC TAA CAC ATG CAA GTC-3′) developed by Marchesi *et al*. (1998) were used for 16S rRNA gene fragment amplification, with a targeted fragment of 1300 bp. The polymerase chain reaction (PCR) mixture consisted of 25 μL of GoTaq Green Mastermix 2X (Promega, Madison, WI, USA), 2 μL of DNA template (~100 ng/μL), 5 μL of 63F primer (10 pmol), and 5 μL of 1387R primer (10 pmol) and adjusted with nuclease-free water to 50 μL. PCR was performed using an initial denaturation at 94°C for 5 min, followed by 30 cycles at 94°C for 30 s, 55°C for 45 s, 72°C for 1 min and 30 s, and post-PCR 4°C for 5 min. The amplified 16S rRNA gene was sequenced in FirstBase, Malaysia. The sequences were compared to the other 16S rRNA sequences in the GenBank National Center for Biotechnology Information (NCBI) database (http://ncbi.nlm.nih.gov) using BlastN (Basic Local Alignment Search Tool) to identify the nearest-neighbour species. The phylogenetic tree was constructed in Molecular Evolutionary Genetics Analysis programme version 11 using the neighbor-joining method with 1000X bootstrap value. All of the 16S rRNA sequences of the two selected bacteria were deposited into GenBank NCBI with accession number MN388834.1, and MN388836.1.

### Extraction and Identification of Metabolites Derived from The Most Promising Isolates

Three-day-old bacteria cultured in seawater complete medium (SWC) medium were mixed with ethyl acetate (1:1, v/v) and shaken continuously for 15 min. Afterward, the solvent layer was separated and evaporated in a rotary evaporator at 40°C. The chemical composition of that extract was then analysed using a gas chromatography-mass spectrometry (GC-MS) instrument (Agilent Technologies 6890N inert C, USA). MSD ChemStation Data Analysis software (G1701EA E.02.02.1431) was used for mass spectra and chromatogram analysis.

### Statistical Analysis

Survival rate of shrimp in pathogenicity test and *in vivo* challenge test were subjected to one-way analysis of variance (ANOVA) shadowed by Duncan’s multiple range test. Statistical significance was established at a probability value of *p* < 0.05. All statistical analysis were performed using Statistical Package for the Social Sciences (SPSS) software version 11.5 for Windows.

## RESULTS

### Non-Pathogenic Characteristic of Sponge-Associated Bacteria on Shrimps

**Figure 1:**
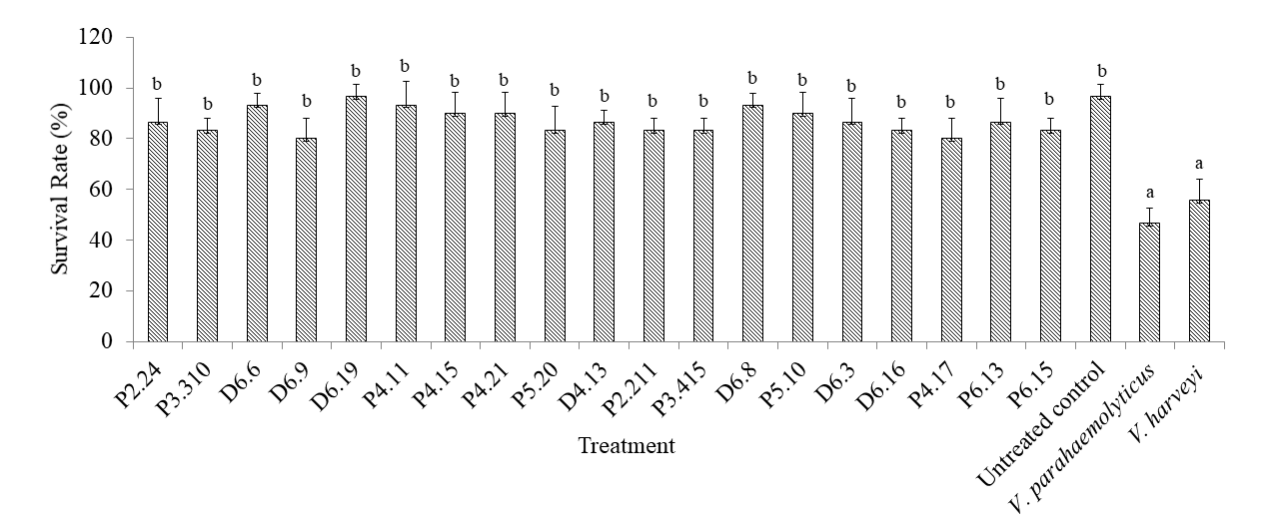
Survival rate of shrimps 7 days after inoculation with sponge-associated bacteria (10^6^ cells/mL) compared to the control infected with *Vibrio* species (10^5^ cells/mL) and untreated control. The values are mean ± standard deviation. Different letters indicate significant differences (*p* < 0.05).

The SR of shrimps after inoculation with the bacterial candidate ranged from 80% to 93.33%, which is relatively not significantly different with the untreated control of approximately 97%. By contrast, more than 50% of shrimps died after inoculation with the respective *V. parahaemolyticus* and *V. harveyi* (Fig. 1).

### LC_50_ Value of *V. harveyi* and *V. parahaemolyticus*

Both *V. harveyi* and *V. parahaemolyticus* exhibited the same LC_50_ value of 10^5^ cells/mL. In this cell density, about 50% of the shrimp population died after 7 days of *Vibrio* inoculation. The value was then used for the challenge test.

### Effect of Sponge-Associated Bacteria Inoculation on the Survival Rate (SR) of Shrimps Infected with *Vibrio* spp

**Figure 2:**
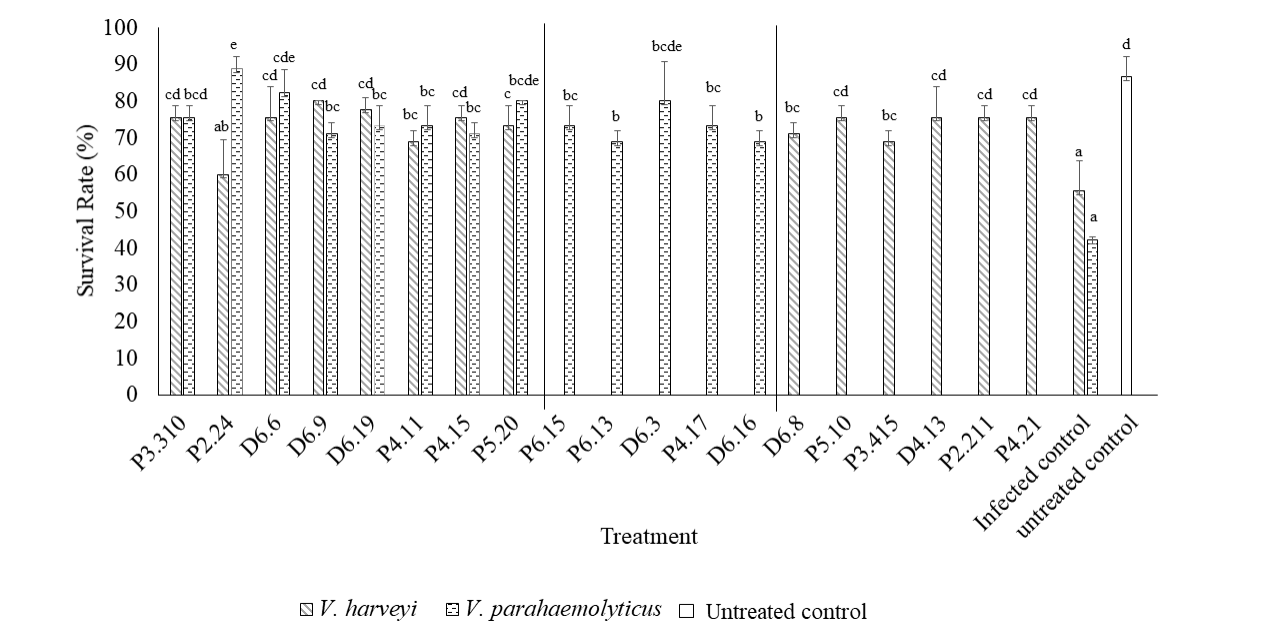
Survival rate of shrimps co-inoculated with sponge-associated bacteria and *Vibrio* species compared to the infected and untreated controls. The values are mean ± standard deviation. Different letters show significant differences (*p* < 0.05).

As shown in Fig. 2, the inoculation of the tested sponge-associated bacteria has dramatically influenced the survival rate (SR) of shrimps infected with each *Vibrio* species. Of the 19 isolates tested, the SR of shrimps increased from 60% to 80% and from 68.89% to 88.89% for shrimps infected with *V. harveyi* and *V. parahaemolyticus*, respectively. Conversely, more than 50% of larvae died after infection with *V. harveyi* (SR = 55.56%) and *V. parahaemolyticus* (SR = 42.22%) species (without bacterial candidate), indicating that the inoculation of these sponge-associated bacteria on the aquarium has improved the survival rate of shrimps, which is not significantly different from the untreated control (SR = 86.67%). The stable *in vivo* anti-*Vibrio* activity have been exhibited by two isolates and coded as D6.9, and P5.20. They could reduce the shrimp mortality caused by *Vibrio* infection and result in a high SR ranging from 71.11% to 80%, which is more than 30% higher than that of the infected control.

### Molecular Identity of the Two Selected Bacteria by 16S rRNA Sequences

**Figure 3:**
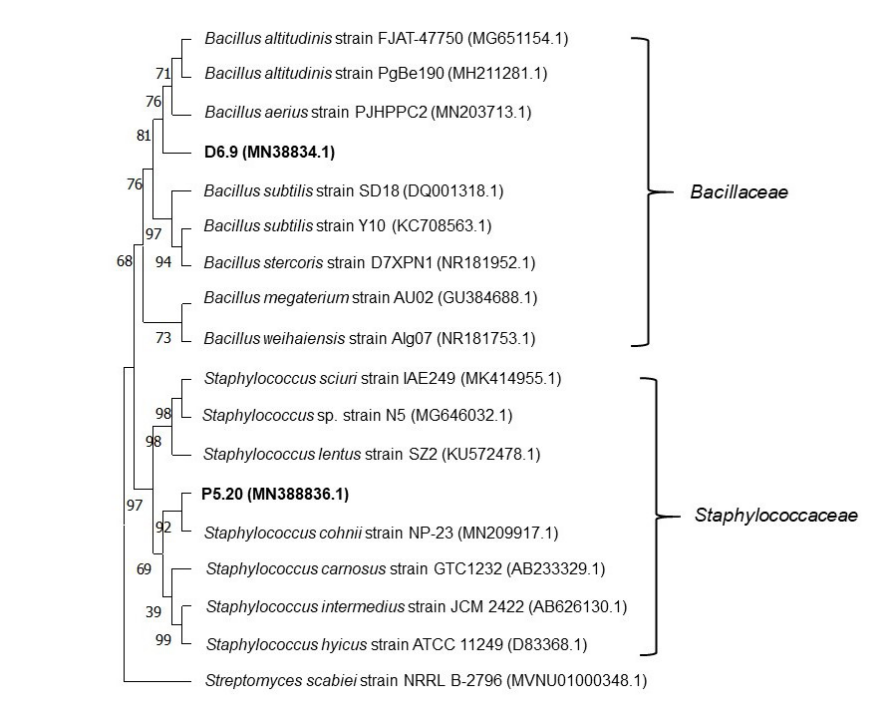
Genetic relationship of two potential bacteria with their closest relative strain based on 16S rRNA sequence.

The amplified 16S rRNA sequences of the two potential bacteria have been sequenced and subjected to NCBI BlastN (Table 1). The sequence of bacterial isolates D6.9, and P5.20 showed respective similarities of 99.92%, and 99.91% to the sequence of two different genera of *Bacillus and Staphylococcus*. The sequences of these isolates can be accessed in the NCBI GenBank database under accession numbers MN388834.1 and MN388836.1. Supporting this result, all of these isolates are also located in different clades (Fig. 3).

### Dominant Component of the D6.9-derived Crude Extract

More than 20 components with different abundance, retention times, and similarities have been found in the crude extract of the D6.9 isolate using the computerised library. Of the 20 components, five components have been found as the dominant compounds as shown in Table 2. The D6.9 extract was characterised by a high content of 1-hydroxy-6-(3-isopropenyl-cycloprop-1-enyl)-6-methyl-ceptan-2-one, hexadecanoic acid (CAS), 4-epicyclomusalenone [(24S)-24-methyl-28-norcycloart-25-en-3-one], and 2,4-dimethyl acetoacetanilide.

## DISCUSSION

*Vibrio* spp., particularly *V. harveyi* and *V. parahaemolyticus*, are quite prevalent in aquaculture, which are responsible for shrimp mortality and considerable decrease in shrimp culture productivity. Sponge-associated bacteria have a great potential for searching beneficial microbes because of their important role in protecting their host from pathogenic microbial infection. The candidate biocontrol agent must be non-pathogenic on shrimps. Nineteen isolates tested in this study were characterised as non-pathogenic bacteria, as indicated by the increased SR of shrimps inoculated with these bacteria. It means that the tested bacteria did not cause significant mortality or were insignificantly different compared to the untreated control. By contrast, inoculation of the aquarium with the *V. harveyi* and *V. parahaemolyticus* displayed significant shrimp mortality of more than 50%. The low survival rate of shrimps was likely caused by *Vibrio* infection disturbing the physiological processes in shrimps. However, a microscopic observation on shrimp larvae fitness after treatment was required to determine the physical and physiological conditions of those larvae.

It is common knowledge that infection mechanisms are influenced by the cell density of pathogenic bacteria. In this case, *Vibrio* infection involved a quorum sensing mechanism, where the responsible gene is activated when the number of cell density is sufficient (Kim *et al*. 2018). In the present investigation, the LC_50_ value was determined to obtain the concentration of *Vibrio* cells needed to cause mortality on nearly 50% of the shrimp population. Both *V. harveyi* and *V. parahaemolyticus* exhibited an LC_50_ value of 10^5^ cells/mL. Supporting this result, the preceding paper also concluded that *Vibrio* species had an LC_50_ value of 10^5^ cells/mL (Rini *et al*. 2017; Joshi *et al*. 2014). This cell density was then used for further analysis.

In our previous studies, it was reported that the 19 isolates tested in this research were characterised with *in vitro* anti-*Vibrio* activity (Wahyudi *et al*. 2018; 2019). Consistently, their activity was also shown in the *in vivo* experiment. The inoculation of these sponge-associated bacteria on shrimps markedly improved the SR of shrimps up to 80% in treatment infected with *V. harveyi* and 88.89% in treatment infected with *V. parahaemolyticus*, approximately 30% higher than uninoculated shrimps. These results suggested that these sponge-associated bacteria could stimulate shrimp resistance from *Vibrio* infection. Consequently, the SR has increased. Similar with the results of this study, inoculating shrimp culture with beneficial bacteria as probiotics feed also reduced shrimp mortality caused by pathogenic bacteria (Hossain *et al*. 2013). A competition in the ecological niche between *Vibrio* and the tested bacteria and the ability of the tested bacteria to produce anti-*Vibrio* compounds are possible mechanisms to inhibit *Vibrio* infection on shrimps. These prospective bacteria also possibly have an important role in maintaining water quality by balancing water microbial population. However, the various effects of these bacteria on shrimp SR indicated the chemodiversity of anti-*Vibrio* compounds produced by these bacteria.

Two isolates coded as D6.9 and P5.20 caused a significant improvement in the SR of shrimps infected with both *V. parahaemolyticus* and *V. harveyi*. These isolates were closely related to the two different genera of *Bacillus and Staphylococcus*. These genera are commonly reported as bioactive compound producers against Gram-positive and Gram-negative bacteria. Gao *et al*. (2017) reported that marine *Bacillus* could inhibit 29 *Vibrio* strains. By contrast, pigmented Gram-positive bacteria, marine *Staphylococcus* sp. associated with microalga, have been studied as antimicrobial agents (Leiva *et al*. 2015). In line with this finding, sponge-derived *Staphylococcus* strains also showed specific antibacterial activity against *Pseudomonas putida* (Matobole *et al*. 2017). In addition, other marine bacterium *Staphylococcus lentus* SZ2 also exhibited antibacterial and antibiofilm activities against *Vibrio harveyi* (Hamza *et al*. 2018).

The D6.9 isolate, the most potential isolate, could improve the SR of shrimps up to 80%, which likely could produce anti-*Vibrio* compounds. The crude extract derived from this isolate is dominated by hexadecanoic acid (palmitic acid), which is well known as an antimicrobial compound (Krishnan *et al*. 2016). Other compounds in this extract, 4-epicyclomusalenone [(24S)-24-methyl-28-norcycloart-25-en-3-one], was also reported as antioxidant agent (Kandasamy *et al*. 2014). Other compounds, 1-hydroxy-6-(3-isopropenyl-cycloprop-1-enyl)-6-methyl-heptan-2-one with low similarity (38%) as the most dominant compound. 2,4-dimethyl acetoacetanilide was also found in D6.9-derived extract (similarity 35%). There is no report about the biological activity of these compounds. The low similarity indicated that GC-MS analysis was not fully confident to determine the type of compound compared from the computerised library, indicating the novelty of this compound.

## CONCLUSION

Nineteen isolates derived from Indonesian Sea sponge consistently showed anti-*Vibrio* activity *in vivo*. They could significantly control *Vibrio* infection on shrimps in various activities. The two selected isolates have been identified as *Bacillus* and *Staphylococcus*, which are well reported as antimicrobial agents. Some components, which are biologically active, have also been found in the extract derived from the most potential isolate, i.e., D6.9. This study suggested that these sponge-associated bacteria could be a hotspot for searching for the anti-*Vibrio* agent. Further research is still needed to explore the anti-*Vibrio* potential of these isolates mainly for future formulation and application of that isolate in shrimp farming.

## Figures and Tables

**Table 1:** 16S rRNA sequence identity of two selected bacteria improving the survival rate of shrimps infected with pathogenic *Vibrio* spp.

Isolate code (accession number)	Closest relative strain (accession number)	Similarity (%)	Query cover (%)
D6.9 (MN388834.1)	*Bacillus aerius* strain PJHPPC2 (MN203713.1)	99.92	99
P5. 20 (MN388836.1)	*Staphylococcus cohnii* strain NP-23 (MN209917.1)	99.91	100

**Table 2:** Five dominant components identified in the crude extract of the D6.9 isolate.

No.	Component	Molecular formula	Peak area (%)	Similarity (%)	Bioactivity (references)
1	1-hydroxy-6-(3-isopropenyl-cycloprop-1-enyl)-6-methyl-heptan-2-one	C_14_H_22_O_2_	9.96	38	Unknown
2	Hexadecanoic acid (CAS)	C_16_H_32_O_2_	9.62	95	Antimicrobial (Krishnan *et al*. 2016)
3	Hexadecanoic acid (CAS)	C_16_H_32_O_2_	7.46	90	Antimicrobial (Krishnan *et al*. 2016)
4	4-epicyclomusalenone [(24S)-24-methyl-28-norcycloart-25-en-3-one]	C_30_H_48_O	5.47	94	Antioxidant (Kandasamy *et al*. 2014)
5	2,4-dimethyl acetoacetanilide	C_12_H_15_NO_2_	4.08	35	Antioxidant and anticancer (Jayashree *et al*. 2012), cytotoxic and antitumour (Priya *et al*. 2015)
